# The prodrome of migraine: mechanistic insights and emerging therapeutic strategies

**DOI:** 10.3389/fneur.2024.1496401

**Published:** 2024-11-29

**Authors:** Linli Gao, Fangling Zhao, Yujie Tu, Kaiming Liu

**Affiliations:** ^1^Department of Cardiology, The Second Affiliated Hospital, School of Medicine, Zhejiang University, Hangzhou, China; ^2^Cardiovascular Key Laboratory of Zhejiang Province, Hangzhou, China; ^3^Department of Neurology, The Second Affiliated Hospital, School of Medicine, Zhejiang University, Hangzhou, China; ^4^Department of Neurology, Affiliated Hospital of Shaoxing University, Shaoxing, China

**Keywords:** migraine, prodrome, treatment strategy, headache, biological mechanisms

## Abstract

Migraine is a common clinical chronic neurovascular disease characterized by recurrent, mostly unilateral, moderate or severe, pulsatile headache. It can be divided into four clinical stages: premonitory (prodrome), aura, headache and postdrome. The early warning value of the prodrome in migraine has been largely verified in various studies. In fact, the prodrome of migraine has received increasing attention as it can serve as an ideal therapeutic window for early intervention and effective treatment of migraine. In recent years, the pathophysiological and molecular biological mechanisms in the prodromal stage of migraine have been extensively studied, and great progress has been made in understanding the disease. This review paper aims to provide an overview of recent studies mainly focused on the prodrome of migraine, discuss the biological mechanisms underlying the clinical profile, and reveal novel therapeutic strategies for preventing or blocking migraine onset during its prodrome.

## 1 Introduction

Migraine, a prevalent and highly debilitating neurological condition, is distinguished by heightened reactivity to light and sound, along with unilateral and pulsating headaches (1). It afflicts approximately 15.0% of the global population, making it one of the most pervasive neurovascular afflictions (2). The migraine attack is a multiphasic event and can generally be divided into a series of stages: premonitory (prodrome), aura, headache, and postdrome, each presenting with a spectrum of complex and mutable symptoms. The entire migraine attack may extend up to a week, profoundly disrupting the patient’s routine existence (3). While some individuals with migraine experience an aura that may be followed by a mild headache or no headache at all, the majority of migraine sufferers are characterized by pronounced headaches (4). These headaches are not only the most conspicuous symptom but also the primary contributor to the disability associated with the condition. The headache is underpinned by the activation of the trigeminovascular system, which is characterized by vasodilation and neurogenic inflammation (5). The trigeminovascular pathway originates from the trigeminal ganglion (TG), where primary sensory neurons, upon depolarization, emit a plethora of neuropeptides, including substance P, calcitonin gene-related peptide (CGRP) and pituitary adenylate cyclase-activating polypeptide (PACAP). These neuropeptides are instrumental in the transmission of pain signals (3). The TG’s peripheral axons serve to innervate both intracranial and extracranial vasculature, including those of the dura mater and pia mater, while the central axons project to the trigeminocervical complex (TCC), which encompasses the trigeminal nerve’s caudate nucleus and the upper cervical spinal cord. At the TCC, these axons synapse with second-order neurons that relay nociceptive signals to higher brain centers, including the brainstem, hypothalamus, and thalamus, culminating in a widespread distribution to various cortical regions for centralized processing (6, 7).

Current research suggested that about one-third of patients, regardless of the type of migraine, experienced prodromal symptoms (8). An increasing number of patients with migraine have perceived one or more additional symptoms before the headache, such as fatigue, neck stiffness, mood changes, nausea, photophobia, yawning, etc. These symptoms, lasting from hours to days, cause varying levels of pain to the patients and seriously affect the quality of daily life (1, 9). Therefore, the prodromal symptoms of migraine have received much attention. Notably, the mechanism by which prodrome exists is not well understood, and it is quite challenging to fully comprehend the deep connotation of prodrome. The prodrome has been strongly demonstrated to have a high predictive value for headache attacks and can serve as an ideal therapeutic window for early intervention (10, 11). Thus, it is of great clinical importance to further understanding the detailed profile of the prodrome. This review mainly systematically discusses the latest progress of the prodrome in its biological mechanism, clinical profile, and novel therapeutic strategy for blocking migraine during the prodrome.

## 2 Prevalence and features of prodromal symptoms

In the 3rd edition of the International Classification of Headache Disorders (ICHD-3), the prodrome is clearly defined as “symptoms preceding and forewarning of a migraine attack by 2–48 h, occurring before the aura in migraine with aura and before the onset of pain in migraine without aura.” Although in a small number of patients, their prodromal symptoms appear earlier (more than 48 h before the attack), this definition is applicable to most clinical research projects (12). The prodromal symptoms before migraine attacks are highly heterogeneous, and their phenotypes are complex and multifaceted. The known prodromal symptoms are divided into four categories: Neuropsychiatric symptoms (anxiety, depression, irritability, fatigue and focusing difficulties); sensory symptoms (photophobia, phonophobia, osmophobia and allodynia); autonomic symptoms (bloating, nausea, pallor, constipation, passing urine frequently and thirst); general symptoms (yawning, stiff neck and eye discomfort) (13–16).

To date, the prevalence of prodromal symptoms is subject to considerable variation, reflecting the diverse expression of migraine across individuals. Pioneering work by Blau conceptualized the “complete migraine,” encompassing prodromal, aura, and headache phases, and observed prodromal symptoms in 34% of the 50 migraine patients prior to the headache phase (15). These symptoms include changes in mood, behavior, arousal, appetite, bowel activity or fluid balance. Notably, the incidence was higher in women, with fatigue occurring at 46.5%, misophonia 36.4%, and yawning 35.8%. The co-occurrence of depression and irritability was the most particularly pronounced, suggesting a complex interplay between emotional and physical manifestations. According to the International Headache Society (IHS), Leslie Kelman designed a tertiary care study to compare the differences in the prodrome between IHS migraine 1.1–1.6 and migraine 1.1–1.7 (8). Prodrome frequency was greater in migraine 1.1–1.6 (32.9%) than in migraine 1.1–1.7 (27.0%) (*p* < 0.01). But there was no difference between prodrome mean duration in migraine 1.1–1.6 (6.8 h) and migraine 1.1–1.7 (9.42 h) (*p* < 0.73). The study suggested the characteristics and importance of the prodromal symptoms in different types of migraine. A prospective study was carried out as the first clinical study to analyze the value of premonitory symptoms (PS) and resolution symptoms (RS) for a group of migraine patients (13). In this study, to reduce recall bias, PS and RS that were experienced by migraine patients the day before (or the day after) the headache began were considered as non-headache symptoms. The study showed that the incidence of PS and RS was 84 and 80%, respectively. It has been shown that anxiety, phonophobia, irritability, unhappiness, and yawning were the most common PS, while weakness, tiredness, sleepiness, and difficulty concentrating were the most common RS. There was a significant difference in the consistency of symptoms reported by patients across the three prodromal episodes, with difficulty concentrating noted in 53% of patients as a prodromal symptom across all episodes, and thirst consistently reported only by 6%. These data indicated that PS and RS were prevalent in migraine attacks, even though their clinical manifestations varied. In their systematic review and meta-analysis, Eigenbrodt et al. (14) employed the term ‘prevalence’ for data extracted from population-based samples and ‘relative frequency’ for data obtained from clinic-based samples. They provided a detailed and critical assessment of these data, ultimately revealing significant heterogeneity in the reporting of migraine prodromes across various studies. The review identified numerous limitations that could account for the conflicting results among studies, such as differences in the nature of the study samples (population-based versus clinical samples), lack of uniformity in the definition of prodromal symptoms, absence of standardized assessment tools, recall bias due to retrospective reporting, and uncertainty in research methodologies. In particular, it is really complicated for migraine patients to completely distinguish between aura and prodrome in many cases, which greatly reduces the accuracy of the research. By way of example, in Poland, a large cohort study on migraine found that the frequency of auras (such as visual symptoms and sensory symptoms) reported by migraine patients was significantly higher than in other studies (17). It was speculated that the reason for this phenomenon was that the prodromal symptoms might not be accurate enough and were often confused with the aura symptoms. These limitations indicate the need for more high-quality, standardized research to better understand and describe the prodromal symptoms of migraines.

Migraine can occur at any age, with the highest prevalence during puberty and adolescence (3). Therefore, the prodromal symptoms in children or adolescents are an important area of migraine research. A questionnaire survey found that at least one prodromal symptom was frequently experienced by children and adolescents with migraine, revealing a prodromal phase incidence rate as high as 67% (16), with facial changes occurring at 44%, fatigue at 42% and irritability at 24%. These symptoms emerged as the most prevalent prodromal indicators. Of note, compared to adults, the prevalence of these symptoms is distinct. Thus, the study speculated that face changes (such as pallor and shadows under the eyes) seemed to be unique to children and adolescents, potentially linked to the immature nervous system regulation characteristic of younger individuals. The specific and underlying neural mechanisms of these prodromal symptoms in children or adolescents remain enigmatic due to a dearth of pediatric migraine research (18).

Despite inter-individual variations in the reported prodromal characteristics, severity, and frequency across studies, there was a strong correlation between these early symptoms and subsequent headache onset. In a clinical trial (19) involving 911 patients with eligible prodromal migraine, 77% were able to be identified with the occurrence of events followed by headache within 1–6 h at least 75% of the time, indicating that prodromal symptoms reliably predict migraine attacks. The most reported prodromal symptoms with variable frequency during the screening period of the study were light sensitivity (57%), fatigue (50%), neck pain (42%), sensitivity to sound (34%), and dizziness (28%). A three-month study recruited subjects who reported prodromal symptoms in at least two of three attacks (20). In this study, 82% of the participants noticed prodromal symptoms and correctly predicted 72% of migraine attacks from their electronic diary entries. Of these prodromal symptoms, the most alarming one was feeling tired and weary (72%). Although considerable evidence suggests that prodromal symptoms occurring before a headache can reliably predict a migraine attack, the ability of sufferers to anticipate the onset of headaches based on these symptoms varies individually (21). By increasing their awareness of the prodrome and utilizing the predictive characteristics of prodromal symptoms, a foundation for early intervention and treatment can be established for patients.

The Belgian Migraine Society conducted a large study aimed at further analyzing the correlation between the occurrence of prodromal symptoms and specific migraine types and patients (22). The study found that female patients reported significantly more prodromal symptoms than male patients. Additionally, the number of prodromal symptoms increased with age and showed a similar trend with the duration of the disease. The number of prodromal symptoms was positively correlated with the number of triggering factors reported by patients, symptoms accompanying the migraine attack, factors known to exacerbate the headache, and symptoms following the attack. Based on the data collected, migraine patients with prodrome appear to have more central nervous system changes than those without prodrome. Therefore, better-designed clinical studies are needed to further evaluate the patients with both types of migraines. Leslie Kelman’s study found that IHS 1.1–1.6 and 1.1–1.7 patients with prodrome exhibited specific performance on several variables: more pronounced triggers, more prolonged auras, more distinct headache characteristics, and more prominent post-headache symptoms (8). Therefore, based on these clues, it is speculated that the prodrome may serve as phenotypic markers, identifying migraine patients with prodrome as a distinct clinical subgroup. It is possible that different subgroups are associated with specific clinical manifestations of the disease, genotypes, or responses to treatment (10). Notedly, this conclusion still merits further experimental data in the future for evidence support.

Systematic study and pattern recognition of migraine prodromal symptoms may provide emerging information for understanding the early warning signs of migraine and contribute to increasing awareness of the prodrome. However, conducting prospective studies on prodromal symptoms in clinical practice is highly challenging. The predominant research method, retrospective analysis based on patient self-reports or questionnaires, is prone to recall bias and results in inaccurate outcomes. Integrating smart devices into daily life for continuous monitoring may offer a novel approach to mitigate this bias (21). To bolster the scientific rigor of migraine research, a multimodal approach that includes retrospective and prospective cohort studies, case-crossover studies, and meticulous case data collection is imperative.

## 3 Pathophysiology mechanisms of the prodrome

The series of prodromal symptoms suggests that some brain nerve tissues may have certain types of pre-existing abnormalities (23). Therefore, it is of great importance to elucidate the detailed pathological process, especially its underlying mechanisms, of prodromal symptoms development. This would enhance our understanding of the nature of the changes in brain function at the prodrome and provide a potential therapeutic target for migraine. The heterogeneity of prodromal symptoms indicates that multiple pathogenic mechanisms are involved, and their interactions have been suggested to play a role in the progression process from the prodrome towards the headache period; however, this remains largely unknown (10). Given the inherent difficulty in capturing spontaneous migraine episodes, particularly the initial prodrome, within standard clinical settings, it becomes essential to utilize established artificial migraine triggers to facilitate experimental procedures, such as nitroglycerin, CGRP and PACAP (9, 24). Interestingly, infusion of nitroglycerin can induce not only migraine-like headaches, but also prodromal symptoms. The triggering consistency of the most common prodromal symptoms, such as inattention and fatigue, is high (>66%) (25). Nitroglycerin is an NO donor with a strong vasodilator effect. Given its lipophilicity, nitroglycerin can readily penetrate the central nervous system, especially mediating the release of key neuropeptides, including CGRP and PACAP. This makes nitroglycerin a reliable agent for inducing migraine attacks and prodromal symptoms in experimental models (24).

In recent years, the rapid development of neuroimaging has facilitated the research progress for studying the pathological mechanism of migraine, especially during the prodrome. The appearance of the prodrome is hypothesized to arise from a disruption in the homeostatic balance between cortical and subcortical regions, leading to increased neuronal excitability and sensitivity to noxious stimuli (1). It is worth noting that how the initial activation of migraine nerves occurs is still unknown. Maniyar’s team employed a nitroglycerin-induced migraine model with H215O-labeled positron emission tomography (PET) to delineate the activation patterns during the prodrome. Their finding implicated that the posterior lateral hypothalamus, periaqueductal gray, ventrolateral periaqueductal gray (PAG), dorsal pons, and various cortical areas (including the occipital lobe, temporal lobe, and prefrontal cortex) were activated in the prodrome (26). Marciszewski et al. (27) compared functional magnetic resonance imaging (fMRI) scans of migraine patients and healthy controls during different stages of the migraine cycle and evaluated brainstem changes and functional connectivity after noxious stimulation. They demonstrated that the pain sensitivity of migraine patients increased during the interictal period of headache, but decreased dramatically within 24 h before the onset of headache. The signal intensity in the spinal trigeminal nucleus region of the migraine patients was significantly higher than that in the control group after receiving noxious stimulation in the prodrome. The dysfunction of the pain regulation circuit reveals that the brainstem plays an important role in the entire migraine cycle. In a double-blind randomized placebo-controlled functional imaging study utilizing pseudo-continuous arterial spin labeling (28), migraine patients who experienced spontaneous prodromal symptoms were selected as experimental subjects. Complete changes in cerebral blood flow maps were obtained during the induction of prodromal symptoms by nitroglycerin. The results conveyed that cerebral blood flow (CBF) increased markedly not only in the hypothalamus but also in the anterior cingulate cortex, audate, midbrain, lentiform, amygdala and hippocampus. These areas are involved in affective, sensory and homeostatic processing. This finding has further bridged clinical manifestations with imaging findings in the prodrome, revealing underlying neurobiological mechanisms in the early stages of migraine. Moreover, the emerging imaging technology of arterial spin labeling holds promise as a vital diagnostic tool in migraine assessment.

These prodrome neuroimaging signature highlights the role of brain activation, particularly activation of the hypothalamus, in the prodrome of nitroglycerin-triggered migraine. The neuroimaging characteristics indicate that the neural network connection pathways are extremely intricate. The prodrome is not caused by a single brain structural change but rather by a high degree of functional integration of the hypothalamus, thalamus, and neural nuclei between the brainstem (29). The brainstem, including the spinal trigeminal nucleus, the rostral ventromedial medulla (RVM), PAG, nucleus raphe magnus (MRN) and locus coeruleus (LC), serves as a key homeostatic modulator. Its aberrant activation during the prodrome is closely tied to appetite and arousal regulation (30). The thalamic region projects trigeminovascular nociceptive information to areas involved in autonomic nerves, sensory emotions, and cognitive functions for processing (7). Paramount among these is the hypothalamic network, which acts as a core hub in the relationship among the three, connecting peripheral stimulation with central sensitization, and promoting the occurrence of migraine in all stages. The hypothalamus, with its circadian rhythm, feeding and stress regulation centers, exerts significant endocrine functions and releases various neurotransmitters, such as orexin, neuropeptide Y (NPY) and dopamine, which participate in the regulation of sleep–wake states, appetite and sensation. It also transmits noxious stimuli at the level of secondary neurons in the TCC (31, 32). Numerous evidences from neuroimaging techniques support the relationship between the prodrome of migraine and changes in the structure and function of the human brain.

Orexin, a hypothalamic neuropeptide, is associated not only with appetite regulation but also with orchestrating multiple physiological activities, such as circadian rhythm, sleep–wake and pain perception (32). The hypothalamic orexin system is closely related to the occurrence of prodromal symptoms. Dual orexin receptor antagonists and intranasal oxytocin have been clinically tested, with strong evidence suggesting significant beneficial effects regarding its efficacy in treating migraine. However, more work is required to evaluate specific and effective therapy targeting the development stage of the prodrome itself (33). NPY is highly expressed in the cerebral cortex, brainstem, and hypothalamic nuclei, and is believed to play an important role in a variety of physiological processes, including food intake, cognition, epileptic seizure activity, learning, stress sensitivity, and mood. NPY receptors are present in the TG and the caudal trigeminal nucleus of the central nervous system, suggesting their role in the pathophysiology of migraine (33). Akihiro Yamanaka et al. found that the NPY pathway interacted with the orexin system and that NPY and its activated NPY-Y1 receptor might be involved in orexin-induced feeding behavior (34). Both may regulate homeostatic functions and the occurrence of prodromal symptoms, such as food craving, sleep–wake, mood changes and autonomic symptoms (31). Dopamine from the hypothalamus incites prodromal symptoms like fatigue, nausea and yawning. Notably, dopamine receptor agonists can stimulate central hypersensitivity reactions, and migraine patients are overly sensitive to dopamine receptor agonists. Preliminary evidence has suggested that dopamine receptor antagonists are effective in preventing migraine in the prodrome, indicating that dopamine signaling pathways likely play an essential role in this stage of migraines (35). Despite numerous limitations in the studies of the hypothalamus and its peptide systems in the mechanism of the prodrome, the available evidence is sufficient to demonstrate their prominent role in migraine pathogenesis.

The eating-fasting network, the sleep–wake network and the emotion-stress network are all deeply intertwined and converge on the interaction between the hypothalamus and the brainstem (36). Notably, whether migraine originates from the peripheral or central system is still controversial. The abnormal sensitization of the limbic system hinges on the input of peripheral noxious stimuli and the induction of *in vitro* drug injection, while the nonspecific symptoms of the prodrome must be perceived by the central nervous system (37). Therefore, the migraine pathogenesis involves both peripheral and central mechanisms, with peripheral noxious stimuli and central pain perception being functionally closely intercalated. To date, whether using clinical research or imaging data, the biological mechanisms of migraine prodrome remain to be fully elucidated.

## 4 Treatment based on the prodrome of migraine

The primary goal of intermittent acute symptomatic treatment is to control the headache and decrease functional disability, typically administered following the emergence of moderate to severe headache attacks. Standard drugs for acute treatment are analgesics (e.g., paracetamol or acetaminophen); non-steroidal anti-inflammatory drugs (NSAIDs) such as ibuprofen, acetylsalicylic acid, diclofenac or naproxen; and triptans (38, 39). Although these medications are initially prescribed to alleviate headache symptoms, their prolonged use may exacerbate the headache condition. Frequent and excessive reliance on one or more medications intended for acute migraine attacks or other primary headache disorders can result in an escalation of headache frequency, potentially leading to the transformation into chronic headaches. This complex scenario is recognized as medication overuse headache (MOH). Consequently, the management of acute headaches becomes increasingly challenging for individuals suffering from MOH (40). In contrast, continuous preventive treatments aim to lessen the severity, frequency and duration of headaches. These preventive treatments are categorized into traditional non-specific drugs like ergotamine, antidepressants, beta-adrenergic receptor-blocking agents (*β*-blockers), calcium channel blockers and antiepileptics; and novel specific drugs, including anti-CGRP monoclonal antibodies (mAbs) and some small molecule CGRP receptor antagonists (gepants) (3, 41). The two treatments complement each other, reducing the disability rate and the risk of drug overuse, as well as improving quality of life for patients (42). Despite the efficacy of these treatments in a majority of patients, a subset remains unresponsive, suggesting a need for alternative strategies (43). Prodromal symptoms, as highlighted by previous research, offer a potential early warning signal for the impending migraine attack and highlight the underlying neuronal mechanisms. These specific neurotransmitters present a vital entry point for novel therapies that could intervene prior to the onset of headache, potentially alleviating or even averting the outbreak of headache (11). Taking certain types of acute medications during the prodrome has demonstrated therapeutic benefits in migraine management (Table 1).

**Table 1:** Mechanism of action and therapeutic targets of drugs in the prodromal phase of migraine.

Therapeutic targets	Related structures	Types of drugs	Main mechanism	Examples	References
5-HT	Trigeminal ganglion, trigeminal nucleus caudalis, cerebral cortex, cerebellum	Triptans	5-HT1B/1D receptor agonists	Almotriptan, eletriptan, frovatriptan, naratriptan, rizatriptan, sumatriptan, and zolmitriptan.	(46, 47, 53)
		Ergot alkaloid	5-HT1B/1D receptor agonists	DHE	(48, 54)
		Ditans	5-HT1F receptor agonists	LY334370, LY344864 and lasmiditan	(46, 47, 57)
PACAP	Trigeminal ganglion, trigeminal nucleus caudalis, sphenopalatine ganglion, thalamus, hypothalamus, cerebellum	PACAP monoclonal antibody	Inhibitor of PACAP peptide	Lu AG09222, LY3451838	(58, 59)
CGRP	Trigeminal ganglion, trigeminal nucleus caudalis, sphenopalatine ganglion, cerebral dura mater, cerebellar cortex	Gepants	Small molecule CGRP receptor antagonists	Olcegepant, telcagepant, ubrogepant, rimegepant and atogepant	(7, 41, 57)
PGs	Trigeminal ganglion, trigeminal nucleus caudalis, middle cerebral artery, cerebral dura mater	NSAIDs	COX inhibitors	Ibuprofen, aspirin, naproxen, ketoprofen and rofecoxib	(66, 67)
Orexin	Hypothalamus, brainstem	OX1R antagonists	Selective OX1R antagonists	—	(31)
NPY	Hypothalamus, brainstem, trigeminal ganglion	NPY Y1 receptor agonists	NPY receptor agonists	—	(69, 70)
NMDA	Trigeminal ganglion, trigeminal nucleus caudalis, thalamus	NMDAR agonists	NMDAR antagonists	MK-801, memantin, ketamine, kynurenate and derivative	(57, 71, 72)

### 4.1 Triptans

Serotonin (5-hydroxytryptamine, 5-HT) is a neurotransmitter that is extensively found throughout the central nervous system and serves as a peripheral vasoconstrictor (77). Levels of 5-HT and its metabolites change in migraine patients: 5-HT increases during migraine attacks and decreases during the interictal phase of migraines (44, 45). The therapeutic efficacy of 5-HT receptor agonists in migraine is hypothesized to stem from their capacity to induce vasoconstriction, curb neurotransmitter release, and impede the activation of various signaling cascades (46, 47). The heterogeneity of 5-HT receptors not only broadens the spectrum of 5-HT’s functional mechanisms but also enhances the selectivity and precision of 5-HT receptor agonists as therapeutic agents.

Among the pharmacological agents designed to target 5-HT receptors, triptans stand out as a specialized class of drugs for migraine treatment. They are widely acknowledged as the primary treatment option for moderate to severe migraine attacks. The early administration of triptans is associated with favorable outcomes for patients with migraine. The current arsenal includes almotriptan, eletriptan, frovatriptan, naratriptan, rizatriptan, sumatriptan, and zolmitriptan (48). An earlier open-label study involving 20 migraine patients in prodrome conducted a two-phase trial (49). During the first stage (baseline), all participants experienced headache attacks after prodrome, with moderate to severe headache accounting for the largest proportion (51% moderate, 44% severe). During the second stage (naratriptan-treatment phase), among the 63 prodromal episodes reported, 40% progressed to headache. The distribution of headache severity shifted to mild in 44%, moderate in 24%, and severe in 32% of cases. Naratriptan administered in the prodrome appears to have a significant preventive effect. If the headache still persisted, its severity appeared to lessen. In another study, compared to placebo, when rizatriptan ODT was taken early in prodrome, headache disappeared within 2 to 24 h after onset (50). In a two-center randomized pilot study (51), two migraine prevention strategies were evaluated: daily topiramat (Group A) versus frovatriptan during prodrome (Group B). Group B experienced fewer adverse events leading to study withdrawal than Group A (4% vs. 18%). The number of headache episodes decreased significantly in both groups, but the efficacy of the two drugs was not directly compared. Frovatriptan is especially suitable for the preventive treatment of menstrual migraine (MM). Patients with predictable MM dose frovatriptan during perimenstrual have a significantly reduced risk of MM in female migraine patients and a low incidence of drug-related adverse events (52). There was no direct evidence to prove the preventive effect of frovatriptan when being taken in the stage of prodrome. But given its positive performance after perimenstrual administration, scholars will continue to explore the therapeutic possibility of frovatriptan during the prodrome based on this field in the near future.

Thus, triptans seem to hold a place in the prodromal treatment of migraine. Triptans have anti-migraine properties as highly selective 5-HT1B and 5-HT1D receptor agonists. Triptans reduce the release of neuropeptides associated with migraine attacks, such as CGRP, substance P, neurokinin A, and glutamate, thereby inhibiting the activation of the trigeminal vascular system to terminate acute migraine attacks (3, 44, 53).

### 4.2 Dihydroergotamine

Dihydroergotamine (DHE), an ergot alkaloid, has very low oral availability due to its poor gastrointestinal absorption and substantial (>90%) first-pass metabolism of what little drug is absorbed. Clinical administration is mostly by nasal spray and muscular or intravenous injection. Like triptans, it acts as an anti-migraine agent by activating 5-HT receptors, which is mainly manifested by constriction of peripheral blood vessels (54). However, DHE has more side effects that is often manifested as nausea. Due to its side effects, low utilization rate, route of administration, and unstable efficacy, it is often relegated to a second-line therapy option (48).

Massiou et al. (55) conducted a double-blind clinical study with a cross-over experimental design to assess the efficacy of DHE nasal spray versus placebo during the prodrome or aura of migraine. After four treatment sessions, a significant difference was observed, with 36% of patients experiencing headache relief with DHE nasal spray compared to 26% with placebo (*p* < 0.05). Similarly, a large multicenter trial in Switzerland investigated the impact of DHE nasal spray on migraine attack management and the prodrome. A favorable response, defined as pain relief, reduced pain intensity, or shortened pain duration, was reported in 76.8% of patients. Only 18.1% experienced mild side effects, such as local nasal irritation (congestion, burning or stinging), nausea, dizziness and vomiting. Notably, among the 143 patients who administered DHE nasal spray during the prodromal phase, 90 (63%) achieved satisfactory therapeutic outcomes (56).

Both trials demonstrated the feasibility of DHE in treating migraine before the onset of headache. However, these early experiments did not clearly define the temporal boundaries of the prodrome, especially between the prodrome and aura. In the future, more scientific and rigorous research protocols are necessary to thoroughly demonstrate the therapeutic effect of ergot and its derivatives in the prodrome of migraines.

### 4.3 Ditans

Ditans, specific selective 5-HT1F receptor agonists, represent a novel site of action for migraine therapy and possess anti-migraine activity (7). Unlike triptans, ditans do not induce vasoconstriction and have a good profile of vascular side effects. This makes them an attractive option for migraine treatment, particularly for patients with cardiovascular or cerebrovascular risk factors (46, 47). Lastiditan is the only ditan currently available for acute migraine treatment. Accordingly, lastiditan may have a broad scope for testing in preventing the migraine (7, 57). More importantly, further clinical studies are supposed to confirm the efficacy and safety of 5-HT1F receptor agonists and to compare them with triptans.

### 4.4 Targeting the PACAP pathway

Except for the innovative approaches for prodromal intervention, it is speculated that other potential neurovascular targets related to the prodrome may pave the way for migraine therapeutics. PACAP is an endogenous multifunctional peptide that belongs to the glucagon/secretin superfamily of peptides. PACAP has two main functional isoforms, including PACAP38 and PACAP27 (respectively consisting of 38 and 27 amino acids), with the former being more widely expressed in human tissues (58). An experimental study found that after intravenous infusion of PACAP, 72% of patients reported migraine-like headache, and nearly half (48%) of patients reported one or more prodromal symptoms before the headache. In comparison, after CGRP infusion, 63% of patients reported migraine-like attacks, with few reporting prodromal symptoms (9). It is noteworthy that patients who experienced migraine-like attacks after receiving PACAP had significantly more prodromal symptoms (such as nausea, photophobia, and fear of sound) than those who did not report attacks. These reported results suggest, to some extent, a deeper link between the PACAP pathway and the prodrome. Consequently, PACAP might be considered a priority target to enhance treatments during the prodrome of migraine. In a phase 2, double-blind, randomized controlled trial, the effectiveness and safety of intravenous Lu AG09222 (a humanized monoclonal antibody that targets the PACAP-38 ligand) in decreasing the monthly migraine frequency among patients who had not responded to previous preventive treatments were successfully demonstrated (59). Given that the PAC1 receptor monoclonal antibody has been proven ineffective in trials for preventing migraines, Lu AG09222 offers renewed hope as a novel targeted therapy for individuals who have not responded to existing treatments (58). Therefore, the development of a novel PACAP monoclonal antibody is crucial in the treatment of migraine, marking an essential direction for future treatment research.

### 4.5 Gepants

CGRP levels increase in blood, saliva, and tear fluid during spontaneous and experimental migraine attacks, aiding in the objective diagnosis of migraines (60). In a study on salivary CGRP levels during the treatment of acute migraine attacks with rizatriptan (61), it was observed that the salivary CGRP levels in the rizatriptan-responsive population were elevated from prodrome to headache. If rizatriptan successfully treated the migraine, salivary CGRP levels would return to near baseline. Salivary CGRP may serve as a unique biomarker for triptans responders. This study supports for the possibility that CGRP changes before a headache. However, research has still been limited regarding the direct role of CGRP level changes in the manifestation of prodrome.

In a landmark trial by Dodick et al. (19), the efficacy of ubrogepant, a gepant, was compared to placebo in a large-scale, randomized, crossover, multicenter study for patients experiencing prodromal symptoms preceding headache. Among those who reliably experienced prodromal symptoms before headache, 46% of those treated with ubrogepant did not experience moderate or severe headaches within 24 h after taking ubrogepant, compared to only 29% in the placebo group (*p* < 0.0001). Additionally, the incidence of adverse events within 48 h was lower with ubrogepant (17%) than with placebo (12%), underscoring its superiority over placebo. These findings are promising for the potential of gepants in prodromal intervention to prevent migraine attacks.

There are two major classes of drugs that antagonize the CGRP pathway, including mAbs and gepants, which are more used as specific prophylactics (62). Although mAbs are well tolerated and effective in migraine treatment, their slow onset of action typically manifests over several weeks, rendering them less suitable for acute migraine treatment. In contrast, gepants are approved as acute antimigraine medications (3). Presently, the emergence of the second-generation gepants, including ubrogepant, atogepant, and rimegepant, has marked a considerable advancement in migraine prophylaxis. Specifically, atogepant has addressed the hepatotoxicity concerns linked to the initial generation of migraine medications, boasting an enhanced safety profile and a mitigated risk of liver injury (63). Furthermore, gepants are attractive drugs that can be used both for the acute and preventive treatment of migraines (39, 41). Given this dual therapeutic capability, good tolerability profile, and lack of association with medication-overuse headache, gepants have begun to be used in ways that go beyond traditional acute and preventive treatments. Interestingly, both CGRP monoclonal antibodies that target the CGRP receptor and those that target other receptors reduced hypothalamic activation (64). The ongoing development and search for novel mAbs or gepants with acute prodromal treatment efficacy present an exciting frontier in migraine research.

### 4.6 NSAIDs

Various humoral factors, such as plasmakinin, serotonin, histamine and prostaglandins (PGs), are involved in the vascular mechanism of migraine attacks: intracranial vasodilation in the prodromal phase and extracranial vasodilation in the headache phase (65). NSAIDs, first-line acute treatment drugs for mild to moderate migraines, exert their anti-inflammatory and analgesic effects by blocking cyclooxygenase (COX), thereby inhibiting the synthesis of PGs from arachidonic acid. In addition, NSAIDs inhibit the synthesis of PGs in the central nervous system and modulate the metabolism of serotonin and catecholamines, which contributes to bolstering the endogenous anti-nociceptive pathways (66). There are two subtypes of COX: COX-1 is involved in homeostasis, and inhibition of COX-1 can result in gastrointestinal bleeding and ulcers; COX-2 is mainly expressed in inflammatory sites, and inhibition of COX-2 can play anti-inflammatory, analgesic and antipyretic roles. Most of the traditional NSAIDs are non-selective agents, so the new generation of NSAIDs has COX-2 highly selective without reducing the anti-migraine effect, thus avoiding gastrointestinal safety impairment (38). Given their affordability and widespread availability, NSAIDs are often more accessible than triptans. Comparative studies on their anti-migraine efficacy have indicated that NSAIDs are at least as effective as triptans in certain contexts (67). Therefore, it will be indispensable to observe whether NSAIDs affect the incidence of headache after prodromal symptoms.

### 4.7 Other potential therapeutic windows

Additional avenues for therapeutic intervention in migraine are emerging, focusing on the role of neurotransmitters secreted by the hypothalamus, including orexin, NPY, and glutamate (68). These molecules are closely linked to the prodromal symptoms of migraine, and the development of novel drugs modulating their activity holds the potential for transformative early intervention strategies. When released from the hypothalamus, Orexin mainly exerts its effects on regulating appetite and arousal by binding to its G protein-coupled receptors (OX1R and OX2R) (34). Currently, selective OX1R antagonists in preclinical models only reflect blocking middle meningeal artery dilation and pro-nociceptive responses of trigeminal nucleus caudalis (TNC) (31). It is time to emphasize the necessity of OX1R antagonist to be tested in clinical trials. NPY is thought to play a key role in the pathophysiology of the prodromal phase of migraine. Specifically, after intravenous injection of NPY or NPY Y1 receptor agonists, the dura mater-induced neuronal discharge is reduced, which indicates that NPY inhibits dura mater-induced trigeminal nerve activity by activating NPY Y1 receptors. This phenomenon is dose-dependent. Importantly, the study suggests that disorders in the NPY system might be the cause of prodromal symptoms (such as changes in appetite) in patients with migraines (69). Chunxiao Yang et al. generated a comprehensive whole-brain NPY expression map in mice for the first time and found that the expression level of NPY changes in specific brain regions in the setting of migraine. Microinjection of NPY or activation of Y1 receptors in the medial habenula (MHb) reduced nitroglycerin-induced allodynia and anxiety without affecting photophobia. NPY has antinociceptive actions in animal models of migraine (70). Therefore, clinical trials are needed to determine the therapeutic promise of targeting NPY signaling in migraine and NPY Y1 receptor agonists may be future candidates for the treatment of migraine. Glutamate is the most essential excitatory neurotransmitter in the brain, and its receptors can be divided into ionic and metabolic. The N-methyl-D-aspartate receptor (NMDAR), a subtype of glutamate-gated ionic channel, is highly expressed in the trigeminal vascular system and is involved in the physiological mechanism of migraine (71). Antagonists of the NMDAR have demonstrated the capacity to inhibit neuronal firing and mitigate trigeminal vascular responses in animal models. Clinical studies have also hinted at the acute and preventive therapeutic effects of NMDAR antagonists (such as ketamine and memantine) in migraine management. Selective antagonists targeting peripheral NMDAR represent a nascent and intriguing frontier in migraine pharmacotherapy (72). In the pathogenesis of migraine, numerous alternative therapeutic targets are awaiting investigation, including targets of the metabotropic receptors (vasoactive intestinal peptide (VIP), amylin, and adrenomedullin), intracellular targets (nitric oxide (NO), phos- phodiesterase-3 (PDE3) and -5 (PDE5)), and ion channels (potassium, calcium, transient receptor potential (TRP), and acid-sensing ion channels (ASIC)). Though research on these targets has not yet yielded new treatments, human challenge studies utilizing these targets have delivered significant understanding into the intricacies of migraine pathogenesis (73). Moreover, these studies may have paved the way for potentially effective treatments for patients who have shown resistance to conventional therapeutic approaches.

The above outlines several new windows for treatment and highlights them as potential targets for preventing migraine attacks based on preclinical and clinical evidence. The migraine pharmacotherapy strategy is transitioning from a symptom-relief focus to a more precision-targeted approach, yet the development of a variety of promising treatment methods remains a lengthy endeavor (41). It is well-established that traditional approaches to acute and preventive migraine management in come with their own set of benefits and drawbacks. However, a novel strategy that addresses migraine before the onset of headache symptoms harnesses the best of both worlds, mitigating some of their respective shortcomings (3). For individuals with a predictable pattern of migraine attacks, using medication during the high-risk period prior to an attack offers a more tailored treatment approach. This not only lessens the frequency and intensity of migraines but also decreases reliance on acute treatment medications (74). Despite these benefits, this innovative treatment strategy is not without its challenges. Defining the high-risk period can be elusive, potentially leading to an increased risk of MOH. Additionally, the unnecessary use of medication could lead to higher medical expenses and greater utilization of resources. Furthermore, the long-term safety of this treatment protocol remains largely uncharted, posing potential unknown risks to patients (75). Of greater importance, because the treatment effect takes a certain amount of time to manifest, the distinction between prodromal symptoms and headache attacks is crucial for early treatment opportunities. Accurately identifying the prodrome time window is the key to the success of preventive treatment. With the rapid development of Internet of Things applications, technological tools in daily life can be skillfully used to improve the accuracy of judging the prodrome. For example, the perfect combination of virtual reality headsets, portable electroencephalogram (EEG) sensors and smart phones can be used to identify patients with difficult-to-detect prodromal symptoms or triggers, and timely take remedial treatment (76). Notably, the high heterogeneity of prodromal symptoms suggests the necessity for individualized treatment regimens. The prodrome of migraine provides patients with a valuable opportunity to prevent migraine attacks in time. To date, there have been few studies on prodromal prophylaxis, and these studies have limitations: the clinical study data is outdated, the sample size of the studies is small, and the experimental studies are still stuck in phases I and II. In addition, conducting similar studies is complicated because patients with prodromal symptoms must be recruited, the reliability of their symptoms determined, and finally, the efficacy tested. Therefore, there is still a long way to go in investigating the clinical value of prodromal symptoms, making it an engaging field of future research.

## 5 Conclusion

The insufficiency in the diagnosis and treatment of migraines, along with the high cost of therapy, has always plagued migraine sufferers and healthcare professionals. With deepening of the understanding of migraine pathophysiology, treatment options for migraines have greatly improved. As the initial phase of a migraine attack, the pathophysiological mechanisms of the prodrome primarily involve the activation of the hypothalamus, changes in the networks of brain regions associated with pain processing, and the release of various neurotransmitters that mediate the prodromal symptoms. This phase offers a valuable opportunity for treatment. Future pharmacological research should adopt a more holistic approach to studying the complexity of migraine and identify desirable targets for creating new therapeutic strategies to control the disease. The ultimate goal of migraine treatment should go beyond relieving or improving the symptoms and develop a novel therapeutic strategy based on a full understanding of the pathophysiological process and related underlying mechanisms.

## References

[1] Dodick DW. A phase-by-phase review of migraine pathophysiology. Headache. (2018) 58:4–16. doi: 10.1111/head.13300 29697154

[2] Stovner LJ Hagen K Linde M Steiner TJ. The global prevalence of headache: an update, with analysis of the influences of methodological factors on prevalence estimates. J Headache Pain. (2022) 23:34. doi: 10.1186/s10194-022-01402-2 35410119 PMC9004186

[3] Dodick DW. Migraine. Lancet. (2018) 391:1315–30. doi: 10.1016/s0140-6736(18)30478-1 29523342

[4] Headache Classification Committee of the International Headache Society (IHS) The International Classification of Headache Disorders, 3rd Edition. Cephalalgia (2018) 38:1–211. doi: 10.1177/0333102417738202 29368949

[5] Silberstein SD. Migraine. Lancet. (2004) 363:381–91. doi: 10.1016/s0140-6736(04)15440-8 15070571

[6] Gross EC Lisicki M Fischer D Sándor PS Schoenen J. The metabolic face of migraine – from pathophysiology to treatment. Nat Rev Neurol. (2019) 15:627–43. doi: 10.1038/s41582-019-0255-4 31586135

[7] Puledda F Silva EM Suwanlaong K Goadsby PJ. Migraine: from pathophysiology to treatment. J Neurol. (2023) 270:3654–66. doi: 10.1007/s00415-023-11706-1 37029836 PMC10267278

[8] Kelman L. The premonitory symptoms (Prodrome): a tertiary care study of 893 migraineurs. Headache. (2004) 44:865–72. doi: 10.1111/j.1526-4610.2004.04168.x 15447695

[9] Guo S Vollesen ALH Olesen J Ashina M. Premonitory and nonheadache symptoms induced by Cgrp and Pacap38 in patients with migraine. Pain. (2016) 157:2773–81. doi: 10.1097/j.pain.0000000000000702 27842045

[10] Rossi P Ambrosini A Buzzi MG. Prodromes and predictors of migraine attack. Funct Neurol. (2005) 20:185–91. 16483459

[11] Buzzi MG Cologno D Formisano R Rossi P. Prodromes and the early phase of the migraine attack: therapeutic relevance. Funct Neurol. (2005) 20:179–83. 16483458

[12] The International Classification of Headache Disorders. 3rd edition (Beta version). Cephalalgia. (2013) 33:629–808. doi: 10.1177/0333102413485658 23771276

[13] Quintela E Castillo J Muñoz P Pascual J. Premonitory and resolution symptoms in migraine: a prospective study in 100 unselected patients. Cephalalgia. (2006) 26:1051–60. doi: 10.1111/j.1468-2982.2006.01157.x 16919055

[14] Eigenbrodt AK Christensen RH Ashina H Iljazi A Christensen CE Steiner TJ. Premonitory symptoms in migraine: a systematic review and meta-analysis of observational studies reporting prevalence or relative frequency. J Headache Pain. (2022) 23:140. doi: 10.1186/s10194-022-01510-z 36371152 PMC9655795

[15] Schoonman GG Evers DJ Terwindt GM van Dijk JG Ferrari MD. The prevalence of premonitory symptoms in migraine: a questionnaire study in 461 patients. Cephalalgia. (2006) 26:1209–13. doi: 10.1111/j.1468-2982.2006.01195.x 16961788

[16] Cuvellier JC Mars A Vallée L. The prevalence of premonitory symptoms in Paediatric migraine: a questionnaire study in 103 children and adolescents. Cephalalgia. (2009) 29:1197–201. doi: 10.1111/j.1468-2982.2009.01854.x 19811504

[17] Waliszewska-Prosół M Straburzyński M Czapińska-Ciepiela EK Nowaczewska M Gryglas-Dworak A Budrewicz S. Migraine symptoms, healthcare resources utilization and disease burden in a large polish migraine cohort: results from ‘Migraine in Poland’-a Nationwide cross-sectional survey. J Headache Pain. (2023) 24:40. doi: 10.1186/s10194-023-01575-4 37041492 PMC10091674

[18] Førland-Schill A Berring-Uldum A Debes NM. Migraine pathophysiology in children and adolescents: a review of the literature. J Child Neurol. (2022) 37:642–51. doi: 10.1177/08830738221100888 35607281

[19] Dodick DW Goadsby PJ Schwedt TJ Lipton RB Liu C Lu K. Ubrogepant for the treatment of migraine attacks during the Prodrome: a phase 3, multicentre, randomised, double-blind, placebo-controlled, crossover trial in the USA. Lancet. (2023) 402:2307–16. doi: 10.1016/s0140-6736(23)01683-5 37979595

[20] Giffin NJ Ruggiero L Lipton RB Silberstein SD Tvedskov JF Olesen J. Premonitory symptoms in migraine: an electronic diary study. Neurology. (2003) 60:935–40. doi: 10.1212/01.wnl.0000052998.58526.a9 12654956

[21] Pavlovic JM Buse DC Sollars CM Haut S Lipton RB. Trigger factors and premonitory features of migraine attacks: summary of studies. Headache. (2014) 54:1670–9. doi: 10.1111/head.12468 25399858

[22] Amery WK Waelkens J Vandenbergh V. Migraine warnings. Headache. (1986) 26:60–6. doi: 10.1111/j.1526-4610.1986.hed2602060.x 3957654

[23] Schulte LH May A. The migraine generator revisited: continuous scanning of the migraine cycle over 30 days and three spontaneous attacks. Brain. (2016) 139:1987–93. doi: 10.1093/brain/aww097 27190019

[24] Sureda-Gibert P Romero-Reyes M Akerman S. Nitroglycerin as a model of migraine: clinical and preclinical review. Neurobiol Pain. (2022) 12:100105. doi: 10.1016/j.ynpai.2022.100105 36974065 PMC10039393

[25] Karsan N Bose PR Thompson C Newman J Goadsby PJ. Headache and non-headache symptoms provoked by nitroglycerin in migraineurs: a human pharmacological triggering study. Cephalalgia. (2020) 40:828–41. doi: 10.1177/0333102420910114 32164428 PMC7528545

[26] Maniyar FH Sprenger T Monteith T Schankin C Goadsby PJ. Brain activations in the premonitory phase of nitroglycerin-triggered migraine attacks. Brain. (2014) 137:232–41. doi: 10.1093/brain/awt320 24277718

[27] Marciszewski KK Meylakh N Di Pietro F Mills EP Macefield VG Macey PM. Changes in brainstem pain modulation circuitry function over the migraine cycle. J Neurosci. (2018) 38:10479–88. doi: 10.1523/jneurosci.1088-18.2018 30341182 PMC6596255

[28] Karsan N Bose RP O’Daly O Zelaya F Goadsby PJ. Regional cerebral perfusion during the premonitory phase of triggered migraine: a double-blind randomized placebo-controlled functional imaging study using pseudo-continuous arterial spin labeling. Headache. (2023) 63:771–87. doi: 10.1111/head.14538 37337681

[29] Karsan N Goadsby PJ. Biological insights from the premonitory symptoms of migraine. Nat Rev Neurol. (2018) 14:699–710. doi: 10.1038/s41582-018-0098-4 30448858

[30] Holland PR Saengjaroentham C Vila-Pueyo M. The role of the brainstem in migraine: potential brainstem effects of Cgrp and Cgrp receptor activation in animal models. Cephalalgia. (2018) 39:390–402. doi: 10.1177/0333102418756863 29411638

[31] Gollion C De Icco R Dodick DW Ashina H. The premonitory phase of migraine is due to hypothalamic dysfunction: revisiting the evidence. J Headache Pain. (2022) 23:158. doi: 10.1186/s10194-022-01518-5 36514014 PMC9745986

[32] Holland PR. Biology of neuropeptides: Orexinergic involvement in primary headache disorders. Headache. (2017) 57:76–88. doi: 10.1111/head.13078 28485849

[33] Strother LC Srikiatkhachorn A Supronsinchai W. Targeted orexin and hypothalamic neuropeptides for migraine. Neurotherapeutics. (2018) 15:377–90. doi: 10.1007/s13311-017-0602-3 29442286 PMC5935635

[34] Yamanaka A Kunii K Nambu T Tsujino N Sakai A Matsuzaki I. Orexin-induced food intake involves neuropeptide Y pathway. Brain Res. (2000) 859:404–9. doi: 10.1016/s0006-8993(00)02043-6 10719096

[35] Akerman S Goadsby PJ. Dopamine and migraine: biology and clinical implications. Cephalalgia. (2007) 27:1308–14. doi: 10.1111/j.1468-2982.2007.01478.x 17970991

[36] Schulte LH Peng K-P. Current understanding of premonitory networks in migraine: a window to attack generation. Cephalalgia. (2019) 39:1720–7. doi: 10.1177/0333102419883375 31615269

[37] Do TP Hougaard A Dussor G Brennan KC Amin FM. Migraine attacks are of peripheral origin: the debate goes on. J Headache Pain. (2023) 24:3. doi: 10.1186/s10194-022-01538-1 36627561 PMC9830833

[38] Ong JJY De Felice M. Migraine treatment: current acute medications and their potential mechanisms of action. Neurotherapeutics. (2018) 15:274–90. doi: 10.1007/s13311-017-0592-1 29235068 PMC5935632

[39] Puledda F Sacco S Diener HC Ashina M Al-Khazali HM Ashina S. International headache society global practice recommendations for the acute pharmacological treatment of migraine. Cephalalgia. (2024) 44:3331024241252666. doi: 10.1177/03331024241252666 39133176

[40] Diener HC Antonaci F Braschinsky M Evers S Jensen R Lainez M. European academy of neurology guideline on the management of medication-overuse headache. Eur J Neurol. (2020) 27:1102–16. doi: 10.1111/ene.14268 32430926

[41] Pellesi L Do TP Hougaard A. Pharmacological management of migraine: current strategies and future directions. Expert Opin Pharmacother. (2024) 25:673–83. doi: 10.1080/14656566.2024.2349791 38720629

[42] Shapiro RE. Preventive treatment of migraine. Headache. (2012) 52:65–9. doi: 10.1111/j.1526-4610.2012.02242.x 23030534

[43] Krymchantowski AV Bigal ME. Polytherapy in the preventive and acute treatment of migraine: fundamentals for changing the approach. Expert Rev Neurother. (2006) 6:283–9. doi: 10.1586/14737175.6.3.283 16533132

[44] Rushendran R Chitra V Ilango K. Major targets involved in clinical management of migraine. Curr Neurovasc Res. (2023) 20:296–313. doi: 10.2174/1567202620666230721111144 37488760

[45] Johnson KW Phebus LA Cohen ML. Serotonin in migraine: theories, animal models and emerging therapies. Prog Drug Res. (1998) 51:219–44. doi: 10.1007/978-3-0348-8845-5_6 9949863

[46] Neeb L Meents J Reuter U. 5-Ht(1f) receptor agonists: a new treatment option for migraine attacks? Neurotherapeutics. (2010) 7:176–82. doi: 10.1016/j.nurt.2010.03.003 20430316 PMC5084098

[47] de Vries T Villalón CM MaassenVanDenBrink A. Pharmacological treatment of migraine: Cgrp and 5-Ht beyond the Triptans. Pharmacol Ther. (2020) 211:107528. doi: 10.1016/j.pharmthera.2020.107528 32173558

[48] Mayans L Walling A. Acute migraine headache: treatment strategies. Am Fam Physician. (2018) 97:243–51. 29671521

[49] Luciani R Carter D Mannix L Hemphill M Diamond M Cady R. Prevention of migraine during Prodrome with Naratriptan. Cephalalgia. (2000) 20:122–6. doi: 10.1046/j.1468-2982.2000.00030.x 10961768

[50] Cady RK Martin VT Géraud G Rodgers A Zhang Y Ho AP. Rizatriptan 10-mg Odt for early treatment of migraine and impact of migraine education on treatment response. Headache. (2009) 49:687–96. doi: 10.1111/j.1526-4610.2009.01412.x 19472447

[51] Cady RK Voirin J Farmer K Browning R Beach ME Tarrasch J. Two center, randomized pilot study of migraine prophylaxis comparing paradigms using pre-Emptive Frovatriptan or daily Topiramate: research and clinical implications. Headache. (2011) 52:749–64. doi: 10.1111/j.1526-4610.2011.02054.x 22188311

[52] MacGregor EA. A review of Frovatriptan for the treatment of menstrual migraine. Int J Women’s Health. (2014) 6:523–35. doi: 10.2147/ijwh.S63444 24904224 PMC4039425

[53] Hu Y Guan X Fan L Jin L. Triptans in prevention of menstrual migraine: a systematic review with meta-analysis. J Headache Pain. (2013) 14:7. doi: 10.1186/1129-2377-14-7 23565873 PMC3620011

[54] Saper JR Silberstein S. Pharmacology of Dihydroergotamine and evidence for efficacy and safety in migraine. Headache. (2006) 46:S171–81. doi: 10.1111/j.1526-4610.2006.00601.x 17078849

[55] Massiou H. Dihydroergotamine nasal spray in prevention and treatment of migraine attacks: two controlled trials versus placebo. Cephalalgia. (1987) 7:440–1. doi: 10.1177/03331024870070s6195

[56] Jenzer G Bremgartner MF. Dihydroergotamine as a nasal spray in the therapy of migraine attacks. Efficacy and tolerance. Schweiz Rundsch Med Prax. (1990) 79:914–7. 2203127

[57] Tajti J Csáti A Vécsei L. Novel strategies for the treatment of migraine attacks via the Cgrp, serotonin, dopamine, Pac1, and Nmda receptors. Expert Opin Drug Metab Toxicol. (2014) 10:1509–20. doi: 10.1517/17425255.2014.963554 25253587

[58] Pellesi L Ashina M Martelletti P. Targeting the Pacap-38 pathway is an emerging therapeutic strategy for migraine prevention. Expert Opin Emerg Drugs. (2024) 29:57–64. doi: 10.1080/14728214.2024.2317778 38337150

[59] Ashina M Phul R Khodaie M Löf E Florea I. A monoclonal antibody to Pacap for migraine prevention. N Engl J Med. (2024) 391:800–9. doi: 10.1056/NEJMoa2314577 39231342

[60] Kamm K. Cgrp and migraine: what have we learned from measuring Cgrp in migraine patients so far? Front Neurol. (2022) 13:930383. doi: 10.3389/fneur.2022.930383 35968305 PMC9363780

[61] Cady RK Vause CV Ho TW Bigal ME Durham PL. Elevated saliva calcitonin gene-related peptide levels during acute migraine predict therapeutic response to Rizatriptan. Headache. (2009) 49:1258–66. doi: 10.1111/j.1526-4610.2009.01523.x 19788468

[62] Messina R Huessler EM Puledda F Haghdoost F Lebedeva ER Diener HC. Safety and tolerability of monoclonal antibodies targeting the Cgrp pathway and Gepants in migraine prevention: a systematic review and network meta-analysis. Cephalalgia. (2023) 43:3331024231152169. doi: 10.1177/03331024231152169 36786548

[63] Baraldi C Beier D Martelletti P Pellesi L. The preclinical discovery and development of Atogepant for migraine prophylaxis. Expert Opin Drug Discov. (2024) 19:783–8. doi: 10.1080/17460441.2024.2365379 38856039

[64] Basedau H Sturm LM Mehnert J Peng KP Schellong M May A. Migraine monoclonal antibodies against Cgrp change brain activity depending on ligand or receptor target – an Fmri study. eLife. (2022):11. doi: 10.7554/eLife.77146 PMC9126581 35604755

[65] Berde B Fanchamps A. Importance of chemical mediators for the pathogenesis and treatment of Hemicrania. Minerva Med. (1976) 67:1991–8. 1084500

[66] Pardutz A Schoenen J. Nsaids in the acute treatment of migraine: a review of clinical and experimental data. Pharmaceuticals (Basel). (2010) 3:1966–87. doi: 10.3390/ph3061966 27713337 PMC4033962

[67] Rothrock JF. Non-steroidal anti-inflammatory drugs (Nsaids) for acute migraine treatment. Headache. (2010) 50:1635–6. doi: 10.1111/j.1526-4610.2010.01785.x 21198567

[68] Karsan N Goadsby PJ. Intervening in the premonitory phase to prevent migraine: prospects for pharmacotherapy. CNS Drugs. (2024) 38:533–46. doi: 10.1007/s40263-024-01091-2 38822165

[69] Oliveira MM Akerman S Tavares I Goadsby PJ. Neuropeptide Y inhibits the Trigeminovascular pathway through Npy Y1 receptor: implications for migraine. Pain. (2016) 157:1666–73. doi: 10.1097/j.pain.0000000000000571 27023421 PMC4949002

[70] Yang C Gong Z Zhang X Miao S Li B Xie W. Neuropeptide Y in the medial Habenula alleviates migraine-like behaviors through the Y1 receptor. J Headache Pain. (2023) 24:61. doi: 10.1186/s10194-023-01596-z 37231359 PMC10209951

[71] Ye R Kong X Han J Zhao G. N-methyl-D-aspartate receptor antagonists for migraine: a potential therapeutic approach. Med Hypotheses. (2009) 72:603–5. doi: 10.1016/j.mehy.2008.11.037 19168292

[72] Kalatharan V Al-Karagholi MA. Targeting peripheral N-methyl-D-aspartate receptor (Nmdar): a novel strategy for the treatment of migraine. J Clin Med. (2023) 12:2156. doi: 10.3390/jcm12062156 36983158 PMC10055974

[73] Al-Hassany L Boucherie DM Creeney H van Drie RWA Farham F Favaretto S. Future targets for migraine treatment beyond Cgrp. J Headache Pain. (2023) 24:76. doi: 10.1186/s10194-023-01567-4 37370051 PMC10304392

[74] Lipton RB Ailani J Mullin K Pavlovic JM Tepper SJ Dodick DW. Situational prevention: pharmacotherapy during periods of increased risk for migraine attacks. Headache. (2024) 64:859–64. doi: 10.1111/head.14775 38957980

[75] Pellesi L Martelletti P. Situational prevention in migraine: are we doing the right thing? J Headache Pain. (2024) 25:137. doi: 10.1186/s10194-024-01841-z 39174943 PMC11340082

[76] Jagarlapudi A Patil A Rathod DP. A proposed model on merging IOT applications and portable EEGS for migraine detection and prevention. 2021 IEEE International Conference on Distributed Computing, VLSI, Electrical Circuits and Robotics (DISCOVER) (2021), 265–269. doi: 10.1109/DISCOVER52564.2021.9663615

[77] Anderson JJ Lau EM. Pulmonary hypertension definition, classification, and epidemiology in Asia, JACC. (2022) 2, 538–546. doi: 10.1016/j.jacasi.2022.04.008 PMC9823284 36624795

